# The different effects of neighbourhood and individual social capital on health-compromising behaviours in women during pregnancy: a multi-level analysis

**DOI:** 10.1186/s12889-015-2213-4

**Published:** 2015-09-14

**Authors:** Andrea Almeida Tofani, Gabriela de Almeida Lamarca, Aubrey Sheiham, Mario Vianna Vettore

**Affiliations:** National Institute of Cancer, Ministry of Health of Brazil, Praça Cruz Vermelha, 23, Centro - Rio de Janeiro, RJ CEP: 20230-130 Brazil; Institute of Studies in Public Health, Federal University of Rio de Janeiro, Avenida Horácio Macedo, S/N - Próximo a Prefeitura Universitária da UFRJ, Ilha do Fundão - Cidade Universitária, Rio de Janeiro, RJ CEP 21941-598 Brazil; Centre of Studies, Policies and Information on Social Determinants of Health, National School of Public Health, Oswaldo Cruz Foundation, Rua Leopoldo Bulhões, 1480, Manguinhos, Rio de Janeiro, RJ CEP: 21041-210 Brazil; Department of Epidemiology and Public Health, University College London, London, WC1E 6BT UK; Unit of Dental Public Health, School of Clinical Dentistry, University of Sheffield, 19 Claremont Crescent, Sheffield, S10 2TA UK

## Abstract

**Background:**

This study assessed clustering of three health-compromising behaviours and explored the association of neighbourhood and individual social capital with simultaneous health-compromising behaviours and patterns of those behaviours in women in the first trimester of pregnancy (baseline) and during the second and third trimesters of pregnancy (follow-up).

**Methods:**

A longitudinal study was conducted on a representative sample of women recruited in antenatal care units grouped in 46 neighbourhoods from Brazil. Neighbourhood-level measures (social capital and socioeconomic status), individual social capital (social support and social networks) and socio-demographic variables were collected at baseline. Smoking, alcohol consumption and inadequate diet were assessed at baseline and follow-up. Clustering was assessed using an observed to expected ratio method. The association of contextual and individual social capital with the health-compromising behaviours outcomes was analyzed through multilevel multivariate regression models.

**Results:**

Clustering of the three health-compromising behaviours as well as of smoking and alcohol consumption were identified at both baseline and follow-up periods. Neighbourhood social capital did not influence the occurrence of simultaneous health-compromising behaviours. More health-compromising behaviours in both periods was inversely associated with low levels of individual social capital. Low individual social capital predicted smoking during whole pregnancy, while high individual social capital increased the likelihood of stopping smoking and improving diet during pregnancy. Maintaining an inadequate diet during pregnancy was influenced by low individual and neighbourhood social capital.

**Conclusions:**

Three health-compromising behaviours are relatively common and cluster in Brazilian women throughout pregnancy. Low individual social capital significantly predicted simultaneous health-compromising behaviours and patterns of smoking and inadequate diet during pregnancy while low neighbourhood social capital was only relevant for inadequate diet. These findings suggest that interventions focusing on reducing multiple behaviours should be part of antenatal care throughout pregnancy. Individual and contextual social resources should be considered when planning the interventions.

## Background

Improving maternal health is high on the current political agenda of global health, according to the United Nations Millennium Development Goals [1]. Health-compromising behaviours such as smoking, alcohol consumption and inadequate diet are major determinants of the global epidemic of chronic diseases [2]. These behaviours are also causally linked to the onset and complications of pre-existing chronic diseases such as diabetes, hypertension and eclampsia, during the gestational period, which in turn, are associated with maternal mortality [3]. The occurrence of multiple health-compromising behaviours is associated with a higher prevalence and mortality rates of chronic diseases [4]. However, investigations on simultaneous health-compromising behaviours in women during pregnancy period are scarce.

Health-related behaviours during pregnancy, such as use of vitamins, dietary habits, alcohol use and smoking are significantly associated with social support [5–7]. Contextual and individual social capital may increase the ability to enforce/reinforce social norms for positive health behaviours as they reduce psychological distress and increase access to material and emotional resources [8]. In addition, high social support has been considered a determinant of earlier initiation of prenatal care and the number of prenatal care visits [5, 9, 10]. Social capital affects maternal health and well-being and pregnancy outcomes [11–13] and may influence health-related behaviours. Social support during pregnancy reduces the risk of low child body length, birthweight and preterm birth [11]; maternal social capital positively relates to women’s self-rated health during pregnancy and childbirth [13].

Social capital is often conceptualized as a contextual phenomen in the sense that it is a community characteristic reflecting the daily interaction between neighbours that may benefit health through interpersonal trust and norms of mutual aid, promoting collective efficacy and neighbourhood cohesion [14, 15]. Social networks and quality of social relationships such as social support are involved in social capital [16]. Contextual and compositional social capital are interrelated and not mutually exclusive terms. Contextual social capital has been conceptualised and measured using different methods to assess collective characteristic of places. Most measurement approaches have been based on aggregating individual perceptions to a spatial scale. Thus, attributing neighbourhood differences to individual factors may not necessarily imply the absence of, or lack of important place-based processes [17].

Health behaviors are unevenly distributed across population groups and distinct patterns of clustering health behaviors have been identified in children and adolescents [18], adults [19–21] and older adults [22]. The studies on clustering of lifestyle risk factors predominantly assessed the pattern of clustering of smoking, drinking alcohol, diet and physical activity [18–22]. Even though the degree of clustering of the health behaviours varied between studies, the strongest associations have been observed for smoking and drinking alcohol [19] and for smoking, drinking alcohol and inadequate diet [21]. Most of the previous studies on social capital and health-related behaviours in pregnant women were cross-sectional and evaluated single behaviours.

There are no longitudinal studies assessing the patters of simultaneous health-compromising behaviours in pregnant women and the possible role of different kinds of social capital on health-compromising behaviours during pregnancy. Therefore, the objective of this study was to assess clustering of three health-compromising behaviours; smoking, alcohol consumption and low fruit/vegetable intake, in the first trimester of pregnancy and in the last 6 months of pregnancy. The influence of neighbourhood and individual social capital on simultaneous health-compromising behaviours and patterns of health-compromising behaviours were also investigated in the above mentioned periods.

**Fig. 1:**
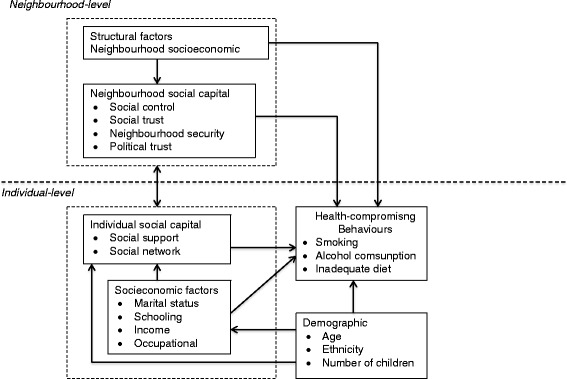
Conceptual model of neighbourhood and individual social capital and behavioural risk factors

The theoretical framework proposed in Fig. 1 encompasses individual and neighbourhood-level factors related to health-compromising behaviours. Structural factors (e.g. area socioeconomic conditions) influences neighbourhood social capital. The neighbourhood-level characteristics are distal determinants of health behaviours, which influence proximate and direct causes of health behaviours. Individual social capital measures such as social support and social networks are important proximate determinants of health behaviours. Demographic and socioeconomic factors influence individual social capital and health behaviours and thus were considered confounding factors.

## Methods

This study was approved by the Committee of Ethics and Research of the Federal University of Rio de Janeiro and informed consent was obtained from all participants.

### Study design and settings

A population-based cohort multilevel study on social capital, health behaviours and health outcomes in a representative sample of pregnant and postpartum women was conducted in two cities in the state of Rio de Janeiro, Brazil. The cities were deliberately selected based on proxy measures for social capital that included violence rates and per capita income [23] and according to demographic indicators. Per capita income for the selected cities with high and low social capital was U$222 and U$101 for city 1 and city 2, while homicides rates was 0.8 (city 1) and 7.4 (city 2) per thousand for the period of study. The population size of the cities was similar (<300,000 inhabitants) and the birth rates were between 130 and 170 births per 100,000 inhabitants [24, 25]. The coverage of antenatal care was greater than 90 % in both cities and antenatal care was provided in few health care units facilitating the recruitment of a representative sample of pregnant women in both cities.

The selection of the sample was conducted at the four main public prenatal care units of the cities where 95 % of the antenatal care in both cities are provided [26].

Pregnant women attending prenatal care in public units are predominantly from moderate and lower social classes. Based on information from the Department of Health of the two cities, pregnant women from 46 neighbourhoods used the antenatal care units selected for this study. Therefore, each woman was allocated to a neighbourhood area, according to residential zip code, which is a good reflection of a neighbourhood geographic area [27].

### Study participants and periods of study

The participants were pregnant women who had sought prenatal care at public health care units administered by the Brazilian National Health Care System. Individual face-to-face interviews were carried out to collect neighbourhood and individual-level primary data between October 2008 and December 2009 [26, 28]. The baseline study was conducted at the antenatal care units during the first trimester of pregnancy and the follow-up data was collected at 30 days postpartum and related to the last 6 months of pregnancy at women’s houses.

### Study power calculation

The formulae for the study power calculation using the method for proportions with cluster randomization [29] was used to estimate the minimum statistical difference between groups considering the observed sample size of 1046 women in 46 neighbourhoods and 23 as the observed average number of women per neighbourhood. The sample intracluster correlation was based on intraclass correlation coefficient of 0.117. The minimum differences of prevalence of cigarette smoking during pregnancy, inadequate diet and alcohol consumption at baseline to be detected between areas with low and high social capital were calculated considering the respective observed prevalences (18.1 %, 53.4 %, 7.6 %). Considering a 80 % power and a significance level of 5 %, the sample size used in this study was able to detect at least 20 % of the differences of the health compromising behaviours.

### Inclusion criteria

The inclusion criteria were women in the first trimester of pregnancy, living at their current address for at least 12 months and who did not change the address by the follow-up study. The two latter criteria were used to assess the effect of social capital on health-compromising behaviours since the neighbourhood effects and individual social capital tend to be stable after some months living in the same place. The interviewers inspected the medical records and all eligible pregnant women were invited to participate. The women were informed about the objectives of the study and their participation was requested before interview. Women who had a miscarriage or abortion were excluded.

### Reliability and consistency

Reliability and internal consistency of the social capital scales were assessed through intraclass correlation coefficient and Cronbach’s α in the test–retest study at 15-day interval. A pilot study (*N* = 130) was performed to test the questionnaires.

The Cronbach’s α coefficient of the social capital questionnaire was 0.684. In the confirmatory factorial analysis, all items of social capital questionnaire loaded coefficients higher than 0.30. Psychometric characteristics of the social capital scales were published elsewhere [28].

### Health-compromising behaviours

The investigated health-compromising behaviours were cigarette smoking, alcohol consumption and inadequate diet. The participants reported current smoking, current alcohol consumption and fruits and vegetable intake at baseline. Frequency of fruits and vegetable intake, smoking and alcoholic beverage consumption during the follow-up period were also recorded. Current smoking was assessed with the following question: ‘Are you a current smoker? (response options: Yes/No)’. The question assessing alcohol consumption was ‘Do you drink alcoholic beverages? (response options: Yes/No)’. These items were tested and used in a study in the same population and shown to be valid [30].

Inadequate diet was evaluated based on the weekly frequency of intake of fruit and vegetables. The items were from the Brazilian national survey on the prevalence of risk factors for chronic diseases in adolescents conducted in 2009 and are considered valid measures of a healthy diet [31]. Pregnant women reporting fruit and vegetable intake lower than five times a week were considered to have inadequate diet [31, 32].

Participants were divided into four groups according to the pattern of each health-compromising behaviour at baseline and follow-up periods. Women who maintained the health-compromising behaviours in both the baseline and follow-up periods were the “stable risk behaviour group”. The “stable healthy behaviour group” included women who did not have the health-compromising behaviours at baseline or follow-up. For example, women who did not smoke throughout gestation. Women who changed their behaviour were classified as “positive behavioural change group” when they changed the behaviour between baseline and follow-up in a positive way, i.e. they stopped smoking during pregnancy. The “negative behavioural change group” were those who adopted the health-compromising behaviour after baseline. For example, adopted a inadequate diet during pregnancy.

### Neighbourhood and individual social capital

Neighbourhood and individual social capital measures were calculated using valid instruments at baseline [33, 34]. Neighbourhood social capital refers to the relationships between social groups and their neighbourhood and is largely based on day-to-day interaction between neighbours [15]. The neighbourhood social capital questionnaire was adapted from a previous study in Brazil and included four dimensions confirmed by factorial analysis: social trust, social control, neighbourhood security and political efficacy [34]. Two core sets of questions from Sampson’s seminal paper on collective efficacy were employed to measure social trust and social control [35] and from Stafford et al. [36]. The social trust measures included from the latter study were if people were comfortable asking a neighbour to collect prescription if ill in bed, to lend a small amount of money or confiding about a personal problem. Included items related to social control were related to people’s reaction if they see children ditching classes, people vandalising things or fighting and treating each other with respect [36]. Items relating to political efficacy were from the American and British Political Action Surveys [37], and frequency of violent episodes in the neighbourhood was used to assess neighbourhood security [35]. As each subscale of the social capital questionnaire consisted of different numbers of items, the final scores for each subscale were standardized from 0 to 100. In this way, subscales were comparable and could be added up to form the neighbourhood social capital variable. The score of social capital was computed at the individual level and then aggregated at neighbourhood level. Participants were grouped into 46 neighbourhood areas: 28 neighbourhoods in City 1 and 18 in City 2. The neighbourhoods were then categorized into three equal groups according to tertiles of the social capital score [29] as follows: low (from 32.08 to 41.63), moderate (from 41.64 to 45.93) and high (from 45.94 to 58.51) neighbourhood social capital. A higher proportion of neighbourhoods in the city with greater violence and low income (city 2) were categorised as low in social capital.

Individual social capital was assessed by the levels of social support and social networks. Social support was measured using a social support scale, which consists of 19 items comprising five dimensions of functional social support: material, affective, emotional, positive social interaction and information [33, 38]. Higher score of social support indicates more support. Social support score was multiplied by 10 on the log scale, so that they indicate a change in the outcome variable for every increase of 10 points in the scale. Social networks are considered as the ‘web’ of social relationships surrounding the individual as well as their characteristics, or groups of people they have contact with [39]. Social networks were assessed based on the number of friends (0–1, 2+) and family members (0–1, 2+) that participants reported that they could talk to openly about any topic.

### Covariates

The covariates were demographic and socioeconomic data collected at baseline.

### Contextual covariate

Social class was evaluated using an economic classification commonly used in Brazil that comprises a group of indicators based on market power and level of education of the head of household [40]. A final score was obtained using a set of points assigned to these indicators which defines the socioeconomic groups; A (highest), B, C, D, and E (lowest). Those with the highest scores represented the highest socioeconomic groups. Because of the small number of observations in classes A and E, data were categorized into three groups: high (A + B); moderate (C); low (D + E). Social class was also aggregated at the neighbourhood level. Neighbourhoods were categorized as low, moderate and high socioeconomic status, based on the tertiles of the distribution of subjects into high social class.

### Individual covariates

Individual maternal socioeconomic and demographic characteristics included marital status (married, living with a partner; has a partner, not living with him; single without a partner), number of children (1 child; 2 children; 3 or more children), years of schooling (0–4; 5–8; 9 or more years), family monthly income (<1 Brazilian Minimal Wage (BMW. One BMW was US$ 178.00 at the time of data collection); 1 or more BMW), occupational context (no paid work – women with no paid work, housewives or unemployed women; paid work – employed women with paid work), age (13–19; 20–30; more than 30 years) and ethnicity. The latter variable was assessed through the self-reported skin colour method as proposed by the Brazilian Institute of Geography and Statistics. Participants were asked to describe their skin colour using the following options: white, brown and black [41].

### Statistical methods

#### Clustering analysis

The clustering of the three health-compromising behaviours before and during pregnancy was examined. Clustering of behaviours existed when the observed combination of behaviours exceeds the expected prevalence of the combination. The expected prevalence of a specific combination of behaviours was calculated on the basis of the individual probabilities of each behaviour based on their occurrence in the study population [19]. The observed/expected ratios were examined by calculating the prevalence odds ratios (POR) and the 95 % confidence interval based on a Poisson distribution [42].

#### Multilevel analysis

This study investigated the association of neighbourhood and individual social capital on health-compromising behaviours before and during pregnancy. The multilevel structure of analysis included 1057 (baseline) and 1046 (follow-up) women (level 1) grouped into 46 neighbourhoods (level 2). A two-level random intercepts and fixed-slopes model structure with individuals nested within neighbourhoods was fitted.

Five outcomes of health-compromising behaviours were considered as follows: (i) number of behaviours at baseline, (ii) number of behaviours at follow-up, (iii) pattern of smoking between baseline and follow-up, (iv) pattern of alcohol consumption between baseline and follow-up, and (v) pattern of diet between baseline and follow-up. Multilevel nested ordered (e.g., number of health related behaviours) and unordered (e.g., pattern of health related behaviours during pregnancy) multinomial logistic regressions, adjusted for confounders were carried out.

Number of health-compromising behaviours was a four-level ordinal outcome, namely none, 1, 2 and 3 risk behaviours, and ordered logit models were used to estimate the cumulative distribution probabilities of the response categories. The reference group was “no health-compromising behaviour”. Coefficients estimated in these models indicated the likelihood of moving into a higher category of number of behaviours. The cumulative response probabilities were modelled, and the proportional odds (cumulative logits) for the three categories presented in relation to independent variables.

“Stable risk behaviour” and “positive behavioural change” were nominal outcomes investigated concerning the patterns of health-related behaviours during pregnancy. They were compared with “stable healthy behaviour” and “stable risk behaviour”, respectively. Unordered logit models were used to estimate the distribution probabilities of each of the response categories.

Fixed- and random parameter estimates for the two-level ordered logit models were calculated by marginal quasi-likelihood (MQL) procedures with first-order Taylor series expansion, RIGLS (restricted iterative generalized least squares) estimation method, as implemented within MLWIN software version 2.24.

The results of multilevel analyses are presented as odds ratios (ORs) with 95 % confidence intervals (95 % CI). In these analyses, variables that presented *P* ≤ 0.10 in bivariate analysis were considered for multivariate analysis. Four models were tested for each outcome. The association between neighbourhood social capital and neighbourhood socioeconomic status (social class) and the health-related behaviours outcomes was tested in Model 1. Individual-level social capital measures (social support and social network) was added in Model 2. Individual-level sociodemographic confounders described in the theoretical model (Fig. 1) were identified from previous studies in pregnant women [9–12]. Socioeconomic factors (marital status, number of children, years of schooling, family monthly income and occupational context) were inserted in Model 3 and demographic characteristics (age and ethnicity) inserted in Model 4. Independent variables of each block were adjusted for each other using backward selection method. Those that remained significant at 5 % (*P* ≤ 0.05) were retained in the analysis for adjustment in the next model.

## Results

Initially, 1750 pregnant women were invited, corresponding to 95 % of women who received prenatal care during the study period. The acceptance rate was 96.2 %. Of the 1684 women interviewed at baseline, 292 were excluded because they moved home during the follow-up or were living at the current address for less than 12 months (*N* = 186), had miscarriages (*N* = 78) and refusal or losses to follow-up (*N* = 28), resulting in 1392 participants. Individuals with missing values for health related behaviours and any independent variable were excluded, which resulted in a final sample of 1057 for the clustering analysis of behaviours at baseline analysis and 1046 for the remaining analysis.

### Description of the study sample

The sample at baseline consisted mostly of pregnant women aged between 20 and 30 years (58.9 %), average 25.17 years. Pregnant women were predominantly, married (70.8 %), with one child (46.7 %). Of the participants, 44.0 % had 5–8 years of schooling, 70.2 % had family income of one minimum wage or more. Low social networks (<2) of relatives and friends were reported by 60.8 and 73.9 % of the sample, respectively. The mean score of social support was 67.9. Mean dimensions of social support scale ranged from 59.5 (material support) to 92.7 (affective support) (Table 1).

**Table 1:** Demographic and socioeconomic characteristics, individual social capital measures and health-compromising behaviours of the sample (*N* = 1046)

	N (%)
Age (years)	
13–19	220 (21.0)
20–30	616 (58.9)
> 30	210 (20.1)
Ethnicity	
White	358 (34.3)
Brown	447 (42.7)
Black	241 (23.0)
Marital Status	
Married, living with partner	741 (70.8)
Has a partner, not living with him	247 (23.6)
Single, without a partner	58 (5.6)
Number of children	
1 child	489 (46.7)
2 children	315 (30.1)
≥ 3 children	242 (23.2)
Years of Schooling	
0 to 4	150 (14.3)
5 to 8	460 (44.0)
≥ 9	436 (41.7)
Family income	
< 1 BMW	312 (29.8)
≥ 1 BMW	734 (70.2)
Occupational context	
No paid work	627 (59.9)
Paid work	419 (40.1)
Social networks of relatives	
< 2	636 (60.8)
≥ 2	410 (39.2)
Social networks of friends	
< 2	773 (73.9)
≥ 2	273 (26.1)
	Mean (SD)
Social support	67.9 ± 15.8
Material support	59.5 ± 21.0
Affective support	92.7 ± 14.1
Emotional support	61.1 ± 20.7
Positive social interaction	64.6 ± 19.4
Information support	61.8 ± 20.0
	N (%)
Smoking (Baseline)	189 (18.1)
Smoking (Follow-up)	114 (10.9)
Alcohol consumption (Baseline)	79 (7.6)
Alcohol consumption (Follow-up)	104 (9.9)
Inadequate diet (Baseline)	558 (53.4)
Inadequate diet (Follow-up)	323 (30.9

Brazilian Minimal Wage (BMW) = US$ 178.00 in 2008

Prevalence of smoking and inadequate diet decreased significantly between baseline and follow-up. The former, from 18.1 % (95 % CI 15.7–20.4) to 10.9 % (95 % CI 9.0–12.8), while the latter from 53.4 % (95 % CI 50.4–56.4) to 30.9 % (95 % CI 28.1–33.7). Alcohol consumption had a non-significant increase between baseline and follow-up; from 7.6 % (95 % CI 6.0–9.2) to 9.9 % (95 % CI 8.1–11.8). Since the limits of 95 % CIs between baseline and follow-up did not overlap for smoking and inadequate diet, these changes were statistically different between the periods (Table 1).

### Clustering of health-compromising behaviours

The observed and expected prevalences of all 8 possible combinations of the three health-compromising behaviours at baseline and follow-up are presented in Table 2. The frequency of women with none, one, two and three health-compromising behaviours behaviours at baseline was 35.1, 50.9, 11.7 and 2.1 %, respectively. These values were 58.7, 32.8, 7.2 and 1.4 % at follow-up. The observed combined prevalence of smoking and alcohol consumption was higher than could have been expected on the basis of the individual probabilities of these two unhealthy behaviours alone at baseline and follow-up. The combination of smoking and alcohol consumption clustered with an O/E ratio of 2.08 (95 % CI: 1.29–3.18) at baseline and 2.67 (95 % CI: 1.76–3.89) at follow-up indicates that the proportion who smoked and drank alcohol at baseline and follow-up were, respectively, 108 % and 167 % greater than the proportion that would be expected had the two health-compromising behaviours occurred independently. The prevalence and prevalence odds ratios (POR) of combinations of the three health-compromising behaviours at baseline and follow-up suggests they are clustered. The proportion with the three health-compromising behaviours at baseline and follow-up were, respectively, 148 % (O/E ratio = 2.48; 95 % CI: 1.67–3.54) and 339 % (O/E ratio = 4.39; 95 % CI: 2.60–6.94) greater than the proportion that would be expected had the three behaviours occurred independently. Once their 95 % CI did not include the value “1”, we can assume these findings were statistically significant.

**Table 2:** Clustering of health-compromising behaviours in pregnant women at 1^st^ trimester of pregnancy (baseline) and during pregnancy (follow-up)

	Baseline (N=1057)							
N Health-compromising behaviours	Smoking	Alcohol consumption	Inadequate diet		Observed prevalence (%)	Expected prevalence (%)	Ratio O/E	95 % CI
0	-	-	-		35.11	34.25	1.03	0.94;1.12
1	+	-	-		7.35	7.93	0.93	0.75;1.11
1	-	+	-		1.78	2.86	0.62	0.39;0.90
1	-	-	+		41.80	40.65	1.03	0.95;1.12
				Total	50.93	51.44	1.00	0.94;1.09
2	+	+	-		1.43	0.66	2.08*	1.29;3.18
2	+	-	+		7.93	9.42	0.84	0.69;1.00
2	-	+	+		2.36	3.40	0.71	0.49;0.99
				Total	11.72	13.48	1.15	0.99;1.33
3	+	+	+		2.14	0.79	2.48*	1.67;3.54
	Follow-up (N=1046)							
0	-	-	-		58.67	56.08	1.05	0.97;1.12
1	+	-	-		5.06	7.22	0.70	0.54;0.90
1	-	+	-		4.05	7.21	0.56	0.42;0.73
1	-	-	+		23.66	23.92	0.99	0.88;1.11
				Total	32.77	38.35	0.86	0.78;0.94
2	+	+	-		2.10	0.78	2.67*	1.76;3.89
2	+	-	+		2.88	3.08	0.92	0.65;1.27
2	-	+	+		2.18	2.57	0.85	0.56;1.22
				Total	7.16	6.43	1.11	0.89;1.36
3	+	+	+		1.40	0.33	4.39*	2.60;6.94

+: health-compromising behaviours present; − : health-compromising behaviours absent; CI = Confidence Interval; * *p* < 0.05

### Social capital and number of health-compromising behaviours

Social capital data and socio-demographic characteristics of the number of health-compromising behaviours groups at baseline and follow-up and crude analysis are presented in Tables 3 and 4. The odds of the number of health compromising behaviours at baseline and follow-up were statistically higher for women with low social networks, low social support and those who were single without a partner. Women with less than 9 years of education and family income of 1 Brazilian Minimal Wage or less, on unpaid work and aged 20 years or less showed significantly higher odds of adopting health compromising behaviours (Tables 3 and 4). The association between Brown ethnicity and number of health compromising behaviours at baseline was marginally statistically significant (Table 3). In the follow-up, women from moderate and low social classes, with three or more children and with Brown and Black ethnicity were more likely to adopt health compromising behaviours (Table 4).

**Table 3:** Distribution of neighbourhood and individual variables and estimated unadjusted odds ratios (OR) for number of risk behaviors groups at baseline (*N* = 1057)

	0	1	2	3	Total	OR^a^	95 % CI	*p*-value
	n (%)	n (%)	n (%)	n (%)	n (%)			
Neighbourhood-level variables								
Neighbourhood social capital								
Low social capital (1st tertile)	121 (31.9)	153 (28.4)	35 (30.2)	7 (30.4)	316 (29.9)	1.31	0.99;1.73	0.058
Moderate social capital (2nd tertile)	112 (29.6)	198 (36.7)	40 (34.5)	10 (43.5)	360 (34.1)	1.02	0.77;1.37	0.870
High social capital (3rd tertile)	146 (38.5)	188 (34.9)	41 (35.3)	6 (26.1)	381 (36.0)	1	1	
Social class								
Low social class (1st tertile)	123 (32.5)	150 (27.8)	36 (31.0)	5 (21.7)	314 (29.7)	1.22	0.92;1.61	0.170
Moderate social class (2nd tertile)	114 (30.1)	188 (34.9)	39 (33.6)	12 (52.2)	353 (33.4)	0.94	0.70;1.25	0.651
High social class (3rd tertile)	142 (37.5)	201 (37.3)	41 (35.3)	6 (26.1)	390 (36.9)	1	1	
Individual -level variables								
Individual social capital								
Social networks								
Relatives								
0–1 relatives	212 (55.9)	340 (63.1)	79 (68.1)	15 (65.2)	646 (61.1)	1.39	1.10;1.77	0.007
2 or more relatives	167 (44.1)	199 (36.9)	37 (31.9)	8 (34.8)	411 (38.9)	1	1	
Friends								
0–1 friends	268 (70.7)	409 (75.9)	88 (75.9)	18 (78.3)	783 (74.1)	1.27	0.97;1.65	0.080
2 or more friends	111 (29.3)	130 (24.1)	28 (24.1)	5 (21.7	274 (21.7)	1	1	
	M (SD)	M (SD)	M (SD)	M (SD)	M (SD)			
Social support (per 10 points)	70.72 (14.06)	67.28 (15.95)	65.63 (16.65)	62.43 (18.46)	67.95 (15.80)	0.82	0.76;0.89	<0.001
Socioeconomic variables								
Marital status								
Married, living with partner	277 (73.1)	384 (71.3)	76 (65.5)	13 (56.6)	750 (71.0)	1	1	
Has a partner, not living with him	85 (22.4)	129 (23.9)	29 (25.0)	5 (21.7)	248 (23.4)	1.13	0.86;1.50	0.372
Single without partner	17 (4.5)	26 (4.8)	11 (9.5)	5 (21.7)	59 (5.6)	2.01	1.20;3.37	0.008
Number of children								
1 child	178 (47.0)	250 (46.4)	55 (47.4)	10 (43.5)	493 (46.6)	1	1	
2 children	126 (33.2)	152 (28.2)	30 (25.9)	10 (43.5)	318 (30.1)	0.88	0.67;1.16	0.384
3 or more children	75 (19.8)	137 (25.4)	31 (26.7)	3 (13.0)	246 (23.3)	1.20	0.90;1.62	0.218
Years of schooling								
0–4 years	45 (11.9)	79 (14.7)	24 (20.7)	3 (13.0)	151 (14.3)	1.88	1.312.70	<0.001
5–8 years	147 (38.8)	248 (46.0)	61 (52.6)	13 (56.5)	469 (44.4)	1.70	1.32;2.20	<0.001
9 years or more	187 (49.3)	212 (39.3)	31 (26.7)	7 (30.4)	437 (41.3)	1	1	
Family income^b^								
0–1 BMW	94 (24.8)	178 (33.0)	37 (31.9)	5 (21.7)	314 (29.7)	1.31	1.01;1.69	0.039
More than 1 BMW	285 (75.2)	361 (67.0)	79 (78.1)	18 (78.3)	743 (70.3)	1	1	
Occupational context								
No paid work	199 (52.5)	333 (61.8)	85 (73.3)	14 (60.9)	631 (59.7)	1.63	1.28;2.07	<0.001
Paid work	180 (47.5)	206 (38.2)	31 (26.7)	9 (39.1)	426 (40.3)	1	1	
Demographic variables								
Age								
13–19	66 (17.4)	121 (22.4)	27 (23.3)	6 (26.1)	220 (20.8)	1.50	1.04;2.16	0.029
20–30	229 (60.4)	311 (57.7)	71 (61.2)	13 (56.5)	624 (59.0)	1.17	0.87;1.58	0.302
More than 30	84 (22.2)	107 (19.9)	18 (15.5)	4 (17.4)	213 (20.2)	1	1	
Ethnicity								
White	148 (39.1)	171 (31.7)	36 (31.0)	8 (34.8)	363 (34.3)	1		
Brown	152 (40.1)	234 (43.4)	56 (48.3)	8 (37.8)	450 (42.6)	1.30	1.00;1.70	0.052
Black	79 (20.8)	134 (24.9)	24 (20.7)	7 (30.4)	244 (23.1)	1.32	0.97;1.80	0.083

^a^OR were estimated using ordered multinomial cumulative logit model. The reference group was ‘No behavioral risk factor’. The coefficients estimated indicated the likelihood of moving into a higher category of the number of risk of behaviors

^b^1 Brazilian Minimal Wage (BMW) = US$ 178.00 in 2008

**Table 4:** Distribution of neighbourhood and individual variables and estimated unadjusted odds ratios (OR) for number of risk behaviors groups at follow-up (*N* = 1046)

	0	1	2	3	Total	OR^a^	95 % CI	*P*-value
	n (%)	n (%)	n (%)	n (%)	n (%)			
Neighbourhood-level variables								
Neighbourhood social capital								
Low social capital (1st tertile)	186 (30.6)	98 (28.1)	22 (29.3)	4 (28.6)	310 (29.6)	1.34	0.98;1.83	0.064
Moderate social capital (2nd tertile)	193 (31.7)	132 (37.8)	27 (36.0)	7 (50.0)	359 (34.3)	1.05	0.76;1.46	0.775
High social capital (3rd tertile)	229 (37.7)	119 (34.1)	26 (34.7)	3 (21.4)	377 (36.1)	1	1	
Social class								
Low social class (1st tertile)	175 (28.8)	107 (30.7)	25 (33.3)	6 (42.8)	313 (29.9)	1.67	1.23;2.26	0.001
Moderate social class (2nd tertile)	185 (30.4)	130 (37.2)	31 (41.4)	4 (28.6)	350 (33.5)	1.50	1.09;2.05	0.012
High social class (3rd tertile)	248 (40.8)	112 (32.1)	19 (25.3)	4 (28.6)	383 (36.6)	1	1	
Individual -level variables								
Individual social capital								
Social networks								
Relatives								
0–1 relatives	351 (57.7)	226 (64.8)	47 (62.7)	12 (85.7)	636 (60.8)	1.35	1.05;1.73	0.018
2 or more relatives	257 (42.3)	123 (35.2)	28 (37.3)	2 (14.3)	410 (39.2)	1	1	
Friends								
0–1 friends	444 (73.0)	261 (74.8)	56 (74.7)	12 (85.7)	773 (73.9)	1.11	0.84;1.47	0.452
2 or more friends	164 (27.0)	88 (25.2)	19 (25.3)	2 (14.3)	273 (26.1)	1	1	
	M (SD)	M (SD)	M (SD)	M (SD)	M (SD)			
Social support (per 10 points)	69.26 (14.64)	67.08 (16.52)	63.10 (17.45)	59.17 (26.25)	67.96 (15.79)	0.87	0.80;0.94	<0.001
Socioeconomic variables								
Marital status								
Married, living with partner	441 (72.5)	247 (70.8)	44 (58.7)	9 (64.3)	741 (70.8)	1	1	
Has a partner, not living with him	142 (23.4)	83 (23.8)	19 (25.3)	3 (21.4)	247 (23.6)	1.11	0.84;1.48	0.468
Single without partner	25 (4.1)	19 (5.4)	12 (16.0)	2 (14.3)	58 (5.5)	2.39	1.44;3.96	<0.001
Number of children								
1 child	296 (48.7)	159 (45.6)	29 (38.7)	5 (35.7)	489 (46.7)	1	1	
2 children	190 (31.3)	99 (28.4)	20 (26.7)	6 (42.9)	315 (30.1)	1.04	0.78;1.38	0.809
3 or more children	122 (20.1)	91 (26.1)	26 (34.7)	3 (21.4)	242 (23.1)	1.54	1.14;2.06	0.005
Years of schooling								
0–4 years	75 (12.3)	57 (16.3)	15 (20.0)	3 (21.4)	150 (14.3)	2.07	1.43;3.00	<0.001
5–8 years	242 (39.8)	172 (49.3)	37 (49.3)	9 (64.3)	460 (44.0)	1.83	1.40;2.39	<0.001
9 years or more	291 (47.9)	120 (34.4)	23 (30.7)	2 (14.3)	436 (41.7)	1	1	
Family income^b^								
0–1 BMW	153 (25.2)	127 (36.4)	28 (37.3)	4 (28.6)	312 (29.8)	1.62	1.25;2.10	<0.001
More than 1 BMW	455 (74.8)	222 (63.6)	47 (62.7)	10 (71.4)	734 (70.2)	1	1	
Occupational context								
No paid work	346 (56.9)	222 (63.6)	49 (65.3)	10 (71.4)	627 (59.9)	1.36	1.06;1.74	0.016
Paid work	262 (43.1)	127 (36.4)	26 (34.7)	4 (28.6)	419 (40.1)	1	1	
Demographic variables								
Age								
13–19	115 (18.9)	85 (24.4)	16 (21.3)	4 (28.6)	220 (21.0)	1.53	1.05;2.23	0.028
20–30	360 (59.2)	202 (57.9)	47 (62.7)	7 (50.0)	616 (58.9)	1.23	0.89;1.69	0.204
More than 30	133 (21.9)	62 (17.8)	12 (16.0)	3 (21.4)	210 (20.1)	1	1	
Ethnicity								
White	237 (39.0)	100 (28.7)	19 (25.3)	2 (14.3)	358 (34.2)	1		
Brown	246 (40.5)	159 (45.6)	34 (45.3)	8 (57.1)	447 (42.8)	1.61	1.22;2.14	0.001
Black	125 (20.6)	90 (25.8)	22 (29.3)	4 (28.6)	241 (23.0)	1.83	1.32;2.54	<0.001

^a^OR were estimated using ordered multinomial cumulative logit model. The reference group was ‘No behavioural risk factor’. The coefficients estimated indicated the likelihood of moving into a higher category of the number of behavioural risk factors

^b^1 Brazilian Minimal Wage (BMW) = US$ 178.00 in 2008

The ordered multinomial logistic multilevel analysis between social capital and the number of health-compromising behaviours at baseline and follow-up are presented in Table 5, respectively. The ordinal model uses cumulative dichotomizations of the categorical outcome. Categories of one, two and three health-compromising behaviours were combined and compared with none risk behaviour. The cumulative response probabilities were modelled, and the proportional odds (cumulative logits) for the three categories presented in relation to neighbourhood and individual variables. In the final models (Model 4), in both baseline and follow-up periods, neighbourhood social capital was not significantly associated with the number of health-compromising behaviours. Of the individual social capital measures, social support remained associated with the number of behaviours in both periods of study in the final models. A 10-point increase in the social support scale (e.g. more supported) reduced the chance of adopting a higher number of health compromising behaviours by 16 % at baseline and 11 % at follow up. Single women, women with low schooling and without paid work had greater odds of a higher number of health-compromising behaviours at baseline in the final model. Covariates statistically associated with the number of health-compromising behaviours in the final model at follow-up were neighbourhood social class, marital status, schooling, age and ethnicity (Table 5).

**Table 5:** Multilevel ordered multinomial regression of the effect of neighbourhood social capital on number of risk factors at baseline (*N* = 1057) and follow-up (*N* = 1046), controlling for individual factors

	Baseline				Follow-up			
	Model 1^b^	Model 2^b^	Model 3^b^	Model 4^b^	Model 1^b^	Model 2^a^	Model 3^b^	Model 4^b^
	OR^a^ (95 % CI)	OR^a^ (95 % CI)	OR^a^ (95 % CI)	OR^a^ (95 % CI)	OR^a^ (95 % CI)	OR^a^ (95 % CI)	OR^a^ (95 % CI)	OR^a^ (95 % CI)
Neighbourhood-level variables								
Neighbourhood social capital								
Low social capital (1st tertile)	1.33 (1.01;1.77)	1.26 (0.96;1.67)	1.32 (0.99;1.75)	1.25 (0.91;1.72)	1.21 (0.89;1.64)	1.19 (0.88;1.61)	1.21 (0.89;1.64)	1.35 (0.97;1.90)
Moderate social capital (2nd tertile)	1.07 (0.79;1.44)	1.03 (0.77;1.38)	1.05 (0.78;1.40)	1.00 (0.73;1.36)	0.98 (0.71;1.35)	0.99 (0.72;1.36)	1.02 (0.74;1.41)	1.18 (0.86;1.62)
Social class								
Low social class (1st tertile)	1.17 (0.88;1.55)				1.45 (1.05;2.01)	1.57 (1.16;2.12)	1.55 (1.15;2.10)	1.51 (1.11;2.07)
Moderate social class (2nd tertile)	0.89 (0.66;1.20)				1.21 (0.89;1.64)	1.41 (1.03;1.94)	1.42 (1.03;1.97)	1.36 (0.97;1.90)
Individual-level variables								
Individual Social Capital								
Social support (per 10 points)		0.84 (0.77;0.90)	0.84 (0.78;0.91)	0.84 (0.77;0.90)		0.89 (0.82;0.96)	0.90 (0.84;0.98)	0.89 (0.82;0.96)
Social network								
0–1 relatives		1.29 (0.97;1.60)				1.26 (0.98;1.63)		
Social network								
0–1 friends		1.11 (0.84;1.46)						
Socioeconomic variables								
Marital status								
Has a partner, not living with him			1.19 (0.90;1.58)	1.12 (0.84;1.51)			1.21 (0.89;1.66)	1.11 (0.81;1.51)
Single without partner			1.95 (1.16;3.28)	1.87 (1.11;3.13)			2.28 (1.36;3.83)	2.30 (1.36;3.86)
Family income								
< 1 BMW^c^			0.94 (0.72;1.23)				1.27 (0.96;1.67)	
Years of schooling								
0–4 years			1.76 (1.22;2.55)	1.85 (1.26;2.71)			1.80 (1.20;2.70)	2.16 (1.50;3.18)
5–8 years			1.64 (1.27;2.13)	1.64 (1.26;2.13)			1.68 (1.27;2.22)	1.81 (1.37;2.39)
Number of children								
2 children							1.06 (0.78;1.44)	
3 or more children							1.22 (0.86;1.72)	
Occupational context								
Without paid work			1.49 (1.16;1.91)	1.43 (1.11;1.83)			1.18 (0.91;1.53)	
Demographic variables								
Age								
13–19				1.40 (0.94;2.10)				1.59 (1.05;2.41)
20–30				1.28 (0.94;1.76)				1.45 (1.04;2.03)
Ethnicity								
Brown				1.18 (0.90;1.55)				1.47 (1.10;1.97)
Black				1.24 (0.90;1.71)				1.66 (1.19;2.33)

Reference categories are in Table 3

^a^OR were estimated using ordered multinomial cumulative logit model. The reference group was ‘Risk factors = 0’. The coefficients estimated indicated the likelihood of moving into a higher category of number of risk behaviors

^b^Variables adjusted for all other variables in the model

^c^1 Brazilian Minimal Wage (BMW) = US$ 178.00 in 2008

### Social capital and patterns of health-compromising behaviours during pregnancy

Neighbourhood and individual social capital were differential characteristics among the patterns of smoking, alcohol consumption and diet between baseline and follow-up periods (Tables 6 and 7). In the crude analysis, low social support was associated with smoking and inadequate diet throughout their pregnancy, and with stopping smoking and positive change in diet during pregnancy. Low social networks of relatives increased the odds of smoking and alcohol consumption during pregnancy, and stopping smoking. Low social networks of friends increased the likelihood of a stable inadequate diet during pregnancy (Table 6). The multivariate multilevel unordered multinomial regression of the effect of neighborhood social capital on patterns of health compromising behaviours is presented in Table 7. Women with lower levels of social support and those with low social networks of relatives had 14 % (OR 0.86 95 % CI 0.77–0.97) and 62 % (OR 1.62 95 % CI 1.02–2.60) higher odds of smoking during all pregnancy (stable smokers) compared to non-smokers. High social support was associated with 27 % higher odds of stopping smoking during pregnancy (OR 1.27 CI 95 % 1.20–1.35) and lower social networks of relatives was associated with 43 % lower odds of stopping smoking during pregnancy (OR 0.57 CI 95 % 0.34–0.93). The different patterns of alcohol consumption were not associated with contextual and individual social capital. Women living in low neighbourhood social capital areas (OR 1.46 95 % CI 1.01–2.11) and low social support (OR 0.98 95 % CI 0.97–0.99) were more likely to have an inadequate diet throughout their pregnancy (stable inadequate diet) compared with those with stable adequate diet. High social support (OR 1.09 95 % CI 1.02–1.18) and low social network of relatives (OR 1.41 95 % CI 1.10–2.06) were significantly associated with a positive change of fruits and vegetable intake during pregnancy. All the results on social capital and patterns of health-compromising behaviours during pregnancy were adjusted for socioeconomic and demographic variables.

**Table 6:** Estimated unadjusted odds ratios (OR) using multilevel unordered multinomial regression for patterns of health compromising behaviurs (*N* = 1046)

	Stable risk behaviour group (Ref: Stable healthy behaviour)			Positive behavioural change (Ref: Stable risk behaviour)		
	Smoking	Alcohol consumption	Inadequate diet	Smoking	Alcohol consumption	Inadequate diet
	OR^a^ (95 % CI)	OR^a^ (95 % CI)	OR^a^ (95 % CI)	OR^a^ (95 % CI)	OR^a^ (95 % CI)	OR^a^ (95 % CI)
Neighbourhood-level variables						
Neighbourhood social capital						
Low social capital (1st tertile)	0.79 (0.48; 1.33)	1.82 (0.77; 4.32)	1.53 (1.08; 2.17)	1.07 (0.60; 1.02)	0.66 (0.30; 1.42)	0.71 (0.52;0.97)
Moderate social capital (2nd tertile)	1.05 (0.66; 1.67)	1.83 (0.79; 4.24)	1.05 (0.72; 1.54)	1.17 (0.69; 1.97)	0.66 (0.31; 1.38)	0.99 (0.72; 1.38)
Social class						
Low social class (1st tertile)	1.35 (0.80; 2.29)	1.65 (0.77; 3.54)	1.69 (1.18; 2.41)	0.52 (0.29; 0.92)	0.66 (0.30; 1.42)	0.58 (0.42; 0.80)
Moderate social class (2nd tertile)	1.79 (1.09; 2.92)	0.84 (0.35; 2.01)	1.39 (0.96; 2.01)	0.51 (0.31; 0.87)	0.67 (0.31; 1.38)	0.61 (0.45; 0.83)
Individual-level variables						
Individual Social Capital						
Social support (per 10 points)	0.98 (0.97; 0.99)	0.98 (0.97; 1.00)	0.98 (0.97; 0.99)	1.02 (1.01; 1.03)	1.00 (0.99; 1.02)	1.13 (1.04; 1.22)
Social network						
0–1 relatives	1.99 (1.26; 3.14)	0.86 (0.44; 1.66)	1.46 (1.08; 1.98)	0.45 (0.29; 0.71)	1.52 (0.79; 2.92)	1.46 (1.11; 1.91)
Social network						
0–1 friends	0.91 (0.58; 1.43)	0.83 (0.40; 1.69)	1.72 (1.21; 2.45)	0.87 (0.53; 1.43)	1.05 (0.53; 2.09)	0.94 (0.69; 1.28)
Socioeconomic variables						
Marital status						
Has a partner, not living with him	1.16 (0.72; 1.86)	1.16 (0.53; 2.52)	0.93 (0.65; 1.33)	0.97 (0.57; 1.65)	1.73 (0.89; 3.36)	1.18 (0.87; 1.61)
Single without partner	2.52 (1.25; 5.08)	1.66 (0.49; 5.68)	2.40 (1.35; 4.26)	0.91 (0.41; 2.02)	1.04 (0.31; 3.58)	0.59 (0.33; 1.07)
Family income						
< 1 BMW^b^	1.06 (0.69;1.64)	0.99 (0.48; 2.03)	2.27 (1.67; 3.08)	0.85 (0.51; 1.40)	0.39 (0.17; 0.94)	0.64 (0.48; 0.85)
Years of schooling						
0–4 years	3.74 (2.05; 6.81)	0.64 (0.14; 3.04)	2.22 (1.44; 3.44)	0.42 (0.22; 0.81)	1.11 (0.41; 3.00)	0.51 (0.34; 0.76)
5–8 years	2.65 (1.60; 4.37)	2.82 (1.30; 60.8)	1.92 (1.38; 2.67)	0.49 (0.30; 0.81)	0.30 (0.15; 0.57)	0.59 (0.44; 0.78)
Number of children						
2 children	1.07 (0.65; 1.75)	1.57 (0.74; 3.34)	0.92 (0.65; 1.31)	0.81 (0.48; 1.38)	0.68 (0.34; 1.35)	0.87 (0.64; 1.18)
3 or more children	1.80 (1.11; 2.90)	1.30 (0.56; 3.04)	1.37 (0.95; 1.97)	0.56 (0.32; 0.98)	0.52 (0.22; 1.25)	0.78 (0.56; 1.08)
Occupational context						
Without paid work	1.20 (0.79; 1.81)	2.11 (0.99; 4.52)	1.92 (1.42; 2.63)	1.14 (0.71; 1.84)	0.41 (0.22; 0.77)	0.85 (0.65; 1.12)
Demographic variables						
Age						
13–19	0.67 (0.35; 1.27)	3.37 (0.92; 12.41)	2.17 (1.37; 3.46)	1.83 (0.92; 3.67)	0.33 (0.14; 0.75)	0.78 (0.52; 1.18)
20–30	0.80 (0.49; 1.33)	2.76 (0.82; 9.26)	1.52 (1.01; 2.27)	1.26 (0.69; 2.31)	0.20 (0.10; 0.43)	0.93 (0.66; 1.31)
Ethnicity						
Brown	1.19 (0.73; 1.93)	1.61 (0.74; 3.50)	1.83 (1.29; 2.61)	0.63 (0.39; 1.93)	0.70 (0.36; 1.39)	0.71 (0.53; 0.96)
Black	1.53 (0.90; 2.60)	1.28 (0.50; 3.28)	1.92 (1.29; 2.87)	0.35 (0.18; 0.68)	0.58 (0.23; 1.45)	0.69 (0.48; 0.97)

Reference categories are in Table 3

^a^OR were estimated using unordered multinomial logit model

^b^1 Brazilian Minimal Wage (BMW) = US$ 178.00 in 2008

**Table 7:** Multivariate multilevel unordered multinomial regression of the effect of neighborhood social capital on patterns of health compromising behaviours (*N* = 1046), controlling for individual factors.

	Stable risk behaviour group (Ref: Stable healthy behaviour)			Positive behavioural change (Ref: Stable risk behaviour)		
	Smoking^b^	Alcohol consumption^b^	Inadequate diet^b^	Smoking^b^	Alcohol consumption^b^	Inadequate diet^b^
	OR^a^ (95 % CI)	OR^a^ (95 % CI)	OR^a^ (95 % CI)	OR^a^ (95 % CI)	OR^a^ (95 % CI)	OR^a^ (95 % CI)
Neighbourhood-level variables						
Neighbourhood social capital						
Low social capital (1st tertile)			1.46 (1.01; 2.11)			1.13 (0.82; 1.59)
Moderate social capital (2nd tertile)			1.19 (0.80; 2.10)			0.83 (0.60; 1.14)
Social class				0.62 (0.36; 1.06)		0.64 (0.46; 0.90)
Low social class (1st tertile)	1.74 (1.03; 2.93)		1.49 (1.03; 2.15)	0.57 (0.34; 0.93)		0.71 (0.52; 0.97)
Moderate social class (2nd tertile)	1.35 (0.77; 2.36)		1.13 (0.77; 1.67)			
Individual-level variables						
Individual Social Capital						
Social support (per 10 points)	0.86 (0.77; 0.97)	0.96 (0.97; 1.00)	0.98 (0.97; 0.99)	1.27 (1.20; 1.35)		1.09 (1.02; 1.18)
Social network						
0–1 relatives	1.62 (1.02; 2.60)		1.06 (0.77; 1.46)	0.57 (0.34; 0.93)		1.41 (1.10; 2.06)
Social network						
0–1 friends			1.21 (0.83; 1.75)			
Socioeconomic variables						
Marital status						
Has a partner, not living with him	1.25 (0.76; 2.06		0.78 (0.53; 1.16)			1.16 (0.85; 1.57)
Single without partner	2.38 (1.16; 4.88)		2.33 (1.30; 4.18)			0.59 (0.32; 1.08)
Family income						
< 1 BMW^c^			1.66 (1.20; 2.30)		0.32 (0.14; 0.70)	0.80 (0.59; 1.07)
Years of schooling						
0–4 years	3.56 (1.91; 6.66)	0.71 (0.15; 3.40)	1.86 (1.14; 3.05)	0.43 (0.21; 0.88)	0.73 (0.36; 1.47)	0.60 (0.40; 0.90)
5–8 years	2.57 (1.54; 4.30)	2.67 (1.22; 5.88)	1.63 (1.14; 2.33)	0.40 (0.24; 0.66)	(0.15 0.08; 0.26)	0.61 (0.49; 0.81)
Number of children						
2 children	1.27 (0.49; 3.27)		1.06 (0.72;1.57)	0.78 (0.45; 1.33)		
3 or more children	1.62 (0.72; 3.65)		1.17 (0.74;1.86)	0.85 (0.44; 1.67)		
Occupational context		1.72 (0.78; 3.78)				
Without paid work			1.47 (1.05; 2.04)		0.35 (0.22; 0.57)	
Demographic variables						
Age						
13–19		2.37 (0.61; 9.23)	2.46 (1.39; 4.35)	1.35 (0.62; 2.92)	0.31 (0.15; 0.65)	
20–30		2.46 (0.71; 8.51)	1.84 (1.20; 2.84)	1.15 (0.64; 2.08)	0.16 (0.09; 0.29)	
Ethnicity						
Brown			1.73 (1.21; 2.48)	0.57 (0.36; 0.92)		0.79 (0.58; 1.07)
Black			1.78 (1.18; 2.69)	0.32 (0.17; 0.61)		0.81 (0.57; 1.15)

Reference categories are in Table 3

^a^OR were estimated using unordered multinomial logit model.

^b^Variables adjusted for all other variables in the model.

^c^1 Brazilian Minimal Wage (BMW) = US$ 178.00 in 2008

## Discussion

This study supports the hypothesis that neighbourhood and individual social capital were predictors for health-compromising behaviours during pregnancy. Different relationships of neighbourhood and individual social capital with simultaneous health-compromising behaviours and different patterns of smoking, alcohol consumption and inadequate diet during pregnancy were found. Pregnant women from neighbourhoods with low contextual social capital were more likely to have inadequate diet throughout the gestation. Individual social capital had significant associations with the number of health-compromising behaviours in both early and late pregnancy. In addition, low individual social capital negatively influenced smoking and inadequate diet throughout pregnancy, whereas high individual social capital was associated with stopping smoking and improving diet during pregnancy.

Personal social resources, namely, individual social capital, of pregnant women seems to be more important for health-compromising behaviours than the place where they live. This finding is consistent with other health outcomes investigated in this sample [13, 22].

The effect of contextual social capital on health-compromising behaviours is disputed. Previous studies on that topic have evaluated individual behavioural factors using cross-sectional design, which limits direct comparisons. To the best of authors’ knowledge, this is the first longitudinal study that evaluated the relationship of neighbourhood and individual social capital with individual health-compromising behaviours as well as the number of behaviours throughout gestation. In some studies, community social capital was a strong predictor of smoking and drinking [43–45]. However, as reported here, previous research found weak associations between neighbourhood social capital and smoking and alcohol consumption [46, 47]. The heterogeneous findings across studies might be explained by variations in the concept and measurement of social capital, the level of aggregation of social capital measures and cultural characteristics of the investigated populations [15]. To overcome the cross-sectional design limitation of previous studies, we assessed multiple and individual risk behaviours in two stages during pregnancy. Although smoking and alcohol consumption were not associated with neighbourhood social capital, inadequate diet throughout pregnancy was predicted by low neighbourhood social capital.

Our findings on the relationship between individual social capital and smoking and inadequate diet agree with previous studies [45–48]. In pregnant women, social support, a proxy measure of individual social capital, was inversely associated with smoking and poor dietary habits [5, 9, 11]. Nevertheless, the previously reported association between individual social capital and alcohol consumption was not found in our study [45, 46, 49]. Stephens [49] argued that the type of social support is a crucial aspect related to alcohol consumption during pregnancy. While pregnancy support protected against alcohol consumption, general support encouraged drinking [49]. The use of a non-specific social support scale in this study might explain the lack of association between social support and alcohol consumption. Another possible explanation for the lack of agreement on the findings is the measure of drinking behaviour.

As expected, the levels of health-compromising behaviours in our representative sample of pregnant women were low compared with that in other populations. Nonetheless, we demonstrated the patterns of clustering health-compromising behaviours in this population in Brazil. Significant clustering of smoking, alcohol consumption and inadequate diet was found in early and late pregnancy. Smoking and alcohol consumption showed the strongest association among the pairwise combinations in both periods. Although this is the first study on clustering health-compromising behaviours in pregnant women, our findings are consistent with previous studies in other population groups, including adults [19–21] and older adults [22].

Even though the evidence from previous studies support our findings, pregnant women have specific characteristics concerning health related issues. They include greater concerns of self-care during pregnancy, more contact with health care professionals and reinforcement of the importance of health-related behaviours during gestational period. The present results support the view that screening for prenatal health-compromising behaviours and interventions to reduce them during prenatal care should not consider each health-compromising behaviour separately. Robust Cochrane systematic reviews demonstrated a significant though modest effect of psychosocial interventions to stop smoking in pregnancy and no impact of home visits during pregnancy on the reduction of alcohol use [50, 51]. There is a need to develop and test comprehensive health promotion approaches to tackle simultaneously health-compromising behaviours in pregnant women since they are strongly related to maternal and new-born’s health.

Although this study uncovers a topic not previously explored in pregnant women using a robust sample, it is not free of limitations. Information on health-compromising behaviours was based on maternal self-reports. Thus, prevalence of smoking and alcohol consumption might have been underestimated since pregnant women are concerned to provide socially acceptable answers to sensitive questions.

Adopting a healthy pregnancy is paramount for maternal and new-born’s health and well-being and health behaviours is of great concern. There is a range of interconnected factors that influences the adoption of healthy behaviours in pregnant women, including family context and its complex structure involving the father of the child and the related social and psychosocial factors [52]. Antenatal care can be considered an unique opportunity to implement health promotion activities to reduce potential risk factors for the maternal diseases and undesirable pregnancy outcomes. In this perspective, a comprehensive prenatal health care approach should consider the family social context in which the pregnant woman is embedded since the social environment plays an important role on the occurrence of multiple behavioural risk factors for maternal and perinatal health.

## Conclusions

Three health-compromising behaviours were relatively common and clustered in the Brazilian women throughout pregnancy. Low individual social capital significantly predicted simultaneous health-compromising behaviours and patterns of smoking and inadequate diet during pregnancy while low neighbourhood social capital was only relevant for inadequate diet. These findings suggest that interventions focusing on the reduction of multiple-behaviours should be part of the antenatal care throughout pregnancy. Individual and contextual social resources should be considered when planning the aforementioned interventions.
